# The Relationship Between Functional Parameters Derived from Diffusion-Weighted MRI and 18F-Fluorodeoxyglucose PET/CT in Head and Neck Squamous Cell Carcinoma: A Systematic Review and Meta-analysis

**DOI:** 10.1055/s-0045-1811201

**Published:** 2025-10-16

**Authors:** Ludovico Maria Garau, Marco Rensi, Livio Bastianutti, Roberto Bologna, Michele Povolato, Decio Capobianco, Fernando Di Gregorio

**Affiliations:** 1 Department of Nuclear Medicine, Santa Maria della Misericordia University Hospital, Udine, Italy

**Keywords:** head and neck squamous cell carcinoma, diffusion-weighted magnetic resonance imaging, positron emission tomography and computed tomography, apparent diffusion coefficient

## Abstract

**Introduction:**

Over the past decade, a mechanistic hypothesis emerged linking limited water diffusivity (often reflecting densely packed, actively dividing tumor cells) to elevated glucose uptake in head and neck cancer.

**Objective:**

A systematic search of MEDLINE via PubMed identified eligible studies assessing the correlation between apparent diffusion coefficients from diffusion-weighted MRI and standardized uptake values from 18F-fluorodeoxyglucose PET/CT in head and neck cancer. Weighted correlation coefficients (ρ) were computed using Fisher Z transformations, and 95% confidence intervals (CIs) were calculated. Heterogeneity was evaluated with Higgins's inconsistency index, and potential publication bias was evaluated by visually inspecting funnel plots.

**Results:**

A total of 25 articles, encompassing 790 patients, were systematically appraised to summarize the available evidence regarding the relationship between functional parameters in head and neck cancer. Thirteen studies, involving 367 patients and reporting a statistically significant inverse correlation between functional parameters, were summarized in the forest plot; the pooled correlation coefficient estimate was ρ = −0.55 (95% CI: −0.624 to −0.473; P < 0.05), with low heterogeneity. A further 12 studies were systematically reviewed for qualitative analysis due to the absence of significant relationships.

**Conclusion:**

The correlations observed in some studies between apparent diffusion coefficients and standardized uptake values may provide insights to develop metrics for evaluating treatment efficacy and predicting clinical outcomes in head and neck cancer. However, not all studies confirmed this result, possibly due to factors such as molecular characteristics or clinical settings.

## Introduction

Head and neck cancers originating from the mucosal epithelium of the oral cavity, oropharynx, nasopharynx, paranasal sinuses, external auditory canal, larynx, and hypopharynx are collectively referred to as head and neck squamous cell carcinoma (HNSCC). 1 Globally, HNSCC is the seventh most common cancer, accounting for 1.9% of all cancer-related deaths, underscoring the urgent need for targeted research and intervention strategies. 2 The National Comprehensive Cancer Network and the European Society for Medical Oncology advocated the integrated use of magnetic resonance imaging (MRI) and 18F-fluorodeoxyglucose (18F-FDG) positron emission tomography/computed tomography (PET/CT) for the diagnostic evaluation of HNSCC. 3 4

Diffusion-weighted (DW) MRI offers both qualitative and quantitative data by measuring the random movement of water molecules within tissues. The apparent diffusion coefficient (ADC), expressed in square millimeters per second, quantifies this water diffusion. In HNSCC, malignant tumors generally exhibit restricted diffusion due to increased cellularity. 5 Combining DW imaging with conventional MRI improves tumor staging, treatment response assessment, and prognosis, establishing DW MRI as a valuable clinical tool. 6

Furthermore, HNSCC often reveals increased glucose uptake at the cellular level. Consequently, 18F-FDG PET/CT is recommended as a staging strategy for subgroups of HNSCC patients at higher risk of metastasis, particularly those in stage III and stage IV. 7 The most common parameter for semi-quantifying metabolic activity is the standardized uptake value (SUV), which represents the ratio of tissue radioactivity uptake to the injected dose, normalized for body weight. Additionally, metabolic tumor volume (MTV), when multiplied by the mean SUV to calculate total lesion glycolysis (TLG), provides volumetric measurements of tumor burden.

Since 2011, increasing attention has been directed toward a biologic-mechanistic hypothesis suggesting a negative correlation between diffusion restriction, reflecting high proliferative activity, and increased glucose metabolism in HNSCC. 8 9 Understanding the relationship between random water molecule displacement and glucose metabolism could enhance knowledge of tumor biology and have clinical implications for early diagnosis, treatment outcome, and treatment planning.

Subsequently, a ratio between ADC and SUV was proposed to account for the interplay between water molecule diffusion and glucose metabolism, demonstrating enhanced accuracy over ADC or SUV alone in distinguishing benign from malignant tumors. 10 More recently, however, contradictory findings have challenged this theory, suggesting that hypercellularity and glucose hypermetabolism may represent distinct and uncorrelated biological phenomena in HNSSC. 11 12

Given conflicting results, this systematic review aims to explore the correlations between ADC and SUV in HNSCC.

## Review of Literature

This study was performed in accordance with and in adherence to the “Preferred Reporting Items for Systematic Reviews and Meta-Analyses” (PRISMA) statement. 13 14 Before starting the literature search, a protocol defining the research question, search methods, inclusion criteria, quality assessment, data extraction, and statistical analysis was developed.

### Search Strategy

The PubMed interface was independently interrogated by two researchers (G. LM. and D. F.) to find relevant published articles that examined the relationship between ADC and SUV in HNSCC.

We used a search algorithm based on a combination of terms, as follows: ((positron emission tomography) OR (PET) OR (positron emission tomography/computed tomography) OR (PET/CT) OR (positron emission tomography-computed tomography)) AND ((18F- FDG) OR (fluorodeoxyglucose) OR (FDG) OR (18FDG) OR (FDG-F18)) AND ((apparent diffusion coefficient) OR (ADC)) AND ((Diffusion Magnetic Resonance Imaging) OR (Diffusion MRI) OR (Diffusion Weighted MRI) OR (DWI) OR (diffusion-weighted magnetic resonance imaging) OR (MRIDWI) OR (diffusion-weighted imaging) OR (diffusion- weighted MRI)). 15 For verification purposes, the following were electronically searched: MEDLINE (through PubMed), Embase (through OVID), Web of Science, Scopus, Cochrane Library, and ClinicalTrials.gov. To expand our search, the references of the retrieved articles were also screened for additional studies.

The search, started on January 1, 2006, was updated until June 31, 2024. The full-text versions of the studies were obtained.

### Study Selection

The included articles fulfilled the following inclusion criteria: (1) full-text papers published in a peer-reviewed scientific journal, focused on untreated patients with HNSCC; (2) index test based on DW MRI and PET/CT (or PET/MRI); (3) histopathology as the reference standard proving HNSCC; (4) sufficient data to retrieve the measure and direction of concordance between ADC and SUV. Moreover, (5) studies with mixed populations (including both untreated and treated patients) were considered for meta-analysis under the following conditions to minimize phenomena that could influence the correlation between ADC and SUV: if the statistical correlation specific to untreated patients could be isolated from treated patients; if the mixed population was enrolled at least 6 weeks after any treatment (to minimize the impact of residual inflammatory phenomena), 16 and lesion segmentation techniques on MRI excluded large vessels (to prevent signal contamination by flowing blood), cystic regions (to avoid non-tumoral fluid artifacts), and necrotic areas (to ensure non-viable tissue did not bias the correlation).

Studies were included in the meta-analysis only if they reported statistically significant relationship between functional parameters, as this was necessary to ensure consistency in the pooled correlation coefficient calculation; conversely, studies were systematically collected only for qualitative analysis if no significant relationship was reported.

### Data Extraction

The data were extracted from the included literatures by two investigators (G. LM. and R. M.) independently, and a standard pro forma was used to compile the following:

a) basic study data (authors, year of publication, country of origin, study design; b) patient characteristics (sample size, gender, median age); c) tumor type (primary HNSSC with or without metastatic lymph nodes); d) tumor anatomical site, such as the oral cavity (including the lips, buccal mucosa, gums, front two-thirds of the tongue, floor of the mouth below the tongue, hard palate, and retromolar trigon); the oropharynx (including the back third of the tongue, soft palate, tonsils, and side and back walls of the throat); the hypopharynx; the rinopharynx; the paranasal sinuses; the larynx; and unusual site, such as external auditory canal; e) staging and grading of the tumors, when possible; f) semi-quantifications of functional parameters derived from 18F-FDG PET/CT and DW MRI (average and maximum values); g) statistical correlations between functional parameters; h) segmentation methods of tumor images used (manual, automatic or semiautomatic); i) technical aspect (vendors) and acquisition protocols.

Extracted data were transferred to a Microsoft Excel spreadsheet (Microsoft, Redmond, WA).

### Quality Assessment

The quality of each study was independently appraised by 3 observers using the QUADAS-2 tool. 17 The QUADAS-2 tool assessed the risk of bias based on 4 domains (patient selection, index test, reference standard, and flow and timing) in terms of risk of bias. Additionally, the first three domains were evaluated for concerns regarding applicability. The provided signaling questions of the QUADAS-2 tool were used to assign judgments of “low”, “high,” or “unclear” risk rating.

### Statistical Analysis

Statistical analysis was carried out by using the MedCalc Statistical Software version 20.1.118 (MedCalc Software, Ostend, Belgium; https:// www. medcalc. org; 2022).

The 95% confidence intervals (CIs) and the weighted summary correlation coefficient between ADC and SUV were calculated by using a Fisher Z transformation of the correlation coefficients. 18 19 Pooled data were presented with 95% confidence interval values (95% CI). A statistical difference of pooled rates was present if there was no overlap among the 95% CI values.

The Higgins' inconsistency index (I ^2^ ) was used to assess data heterogeneity. An I ^2^ index > 50% indicated heterogeneity among studies. 20 The interpretation of heterogeneity was performed at a significance level of P ≤ 0.05. The choice between fixed or random effects models for meta-analytic estimates depended on the degree of inconsistency, with the random effects model selected based on the DerSimonian and Laird method when substantial heterogeneity was present. 21 Publication bias was assessed through visual inspection of funnel plots.

Correlation strength was classified as very weak (ρ ≤ ± 0.19), weak (ρ ± 0.20–0.39), moderate (ρ ± 0.40–0.59), strong (ρ ± 0.60–0.79), very strong (ρ ± 0.80–0.99), and perfect (ρ = ± 1).

## Data Synthesis

### Selection and Characteristics of Studies

**Fig. 1:**
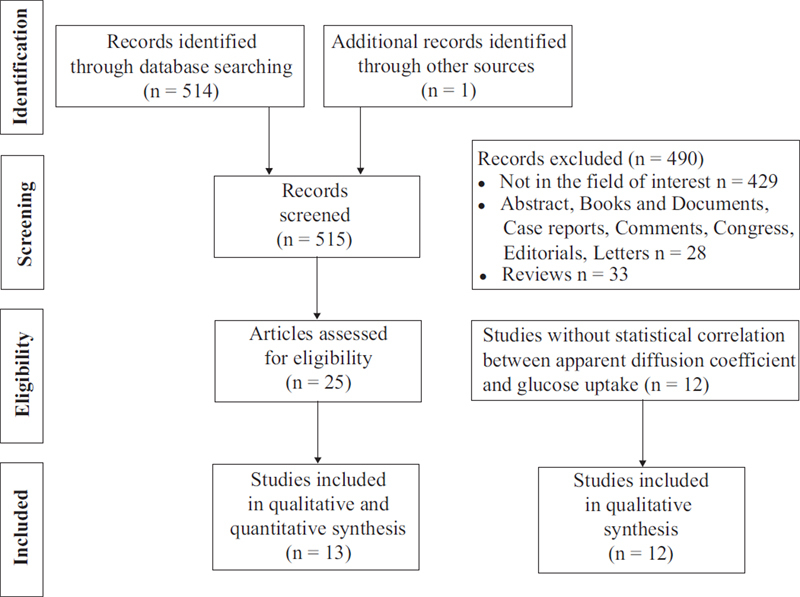
Flowchart detailing the process of inclusion and exclusion of articles in this meta-analysis.

The comprehensive computer literature search revealed 515 articles ( Fig. 1 ). According to the inclusion criteria, twenty-five articles, including 790 patients 8 9 11 12 22 23 24 25 26 27 28 29 30 31 32 33 34 35 36 37 38 39 40 41 42 were systematically appraised to summarize the available evidence concerning the relationship between functional parameters in HNSCC ( Table 1 ). The oropharynx was the most common tumor site, comprising 31% of all HNSCC cases, followed by the oral cavity and hypopharynx (20%), nasopharynx (15%), larynx (11%), and paranasal sinuses (3%).

**Table 1:** Characteristics of studies evaluating the correlation between apparent diffusion coefficient and standardized uptake value in HNSCC

Year	Study	Design (Pros; Retro)	Tumor (T; N)	Oral (No.)	Orop (No.)	Hypo (No.)	Rino (No.)	Sinus (No.)	Laryn (No.)	Other (No.)	Pt (No.)
2011	Fruehwald-Pallamar 8	Pros	T	20	5	3	2	1	0	0	31
2011	Choi 36	Retro	T	32	12	0	0	3	0	0	47
2012	Nakajo 37	Retro	T	3	8	9	1	4	1	0	26
2012	Nakamatsu 38	Retro	N	5	1	12	1	0	4	1	24
2013	Varoquaux 39	Retro	T	6	13	6	3	1	3	2	33
2014	Ng 40	Pros	N	0	37	32	0	0	0	0	69
2015	Covello 41	NA	T	6	4	0	4	3	25	4	44*
2015	Han 42	Retro	T	8	9	4	8	2	2	0	34
2015	Gawlitza 22	Retro	T	5	7	3	0	0	2	0	17**
2015	Martins 23	Pros	T + N §	0	16	2	0	0	4	1	23
2016	Surov 10	Pros	T	NA	NA	NA	NA	NA	NA	NA	11
2017	Núñez 25	Pros	T + N §§	0	5	0	0	0	0	1	6
2017	Leifels 26	Pros	T	7	14	7	0	0	5	0	34
2017	Rasmussen 9	Pros	T	NA	NA	NA	NA	NA	NA	NA	17
2018	Dang 27	Pros	T	11	0	5	4	0	3	0	23
2020	Cheng 28	NA	T	0	0	0	35	0	0	0	35
2020	Çolak 11	Retro	T	4	3	4	14	2	8	1	36
2020	Zhang 29	Pros	T	0	0	9	15	0	3	0	27
2021	Garau 30	Retro	T	5	23	0	8	0	0	0	36***
2021	Paudyal 31	Pros	N	0	22	0	0	0	0	1	23
2021	Bülbül 32	Retro	T	5	2	0	2	0	5	0	14
2022	Gupta 33	Retro	T	NA	NA	NA	NA	NA	NA	NA	20
2022	Freihat 12	Retro	T	22	0	32	0	0	15	0	71
2022	de Koekkoek-Doll 34	Retro	N	19	32	4	2	3	8	10	78
2023	Wongsa 35	Retro	T	5	0	0	3	0	0	1	11

HNSCC, Head and neck squamous cell carcinoma; Hypo, hypopharynx; N, lymphadenopathy from HNSCC; No., number of enrolled patients with HNSCC; Oral, oral cavity; Orop, oropharyngeal; Other, auditory canal, parotid; Pros, prospective study; Pt, patients; Retro, retrospective study; Rino, rinopharynx; T, primary tumor.

* 22 primary tumors and 22 recurrent tumors enrolled 6–8 weeks after the end of surgery (12 patients), radiotherapy (4 patients) or a combination of surgery and radiotherapy (6 patients)

** 6 recurrent cancers enrolled 12 to 120 months (mean time, 46 months) after undefined therapy

*** 17 patients enrolled 137 days (mean interval) after chemo-radiotherapy

§ 23 primary tumors and 52 metastatic lymph nodes were examined from 23 patients

§§ A total of 11 neck nodal metastases and 5 primary tumors were analyzed (3 patients had more than one node and 1 patient had an unknown primary tumor site); NA, not applicable

Tumors with limited extension, not exceeding 2 cm in their greatest dimension, comprised 13% of the sample. Additionally, 26% were classified as T2, 29% as T3, and 32% presented with moderately advanced or very advanced local disease (T4). Metastatic lymph nodes were reported in approximately 45% cases. However, lymph node status and distant metastases were not described in twelve articles. Poorly and well-or-moderately-well differentiated tumors represented 36% and 64% of the sample, respectively.

### Quality Assessment

**Fig. 2:**
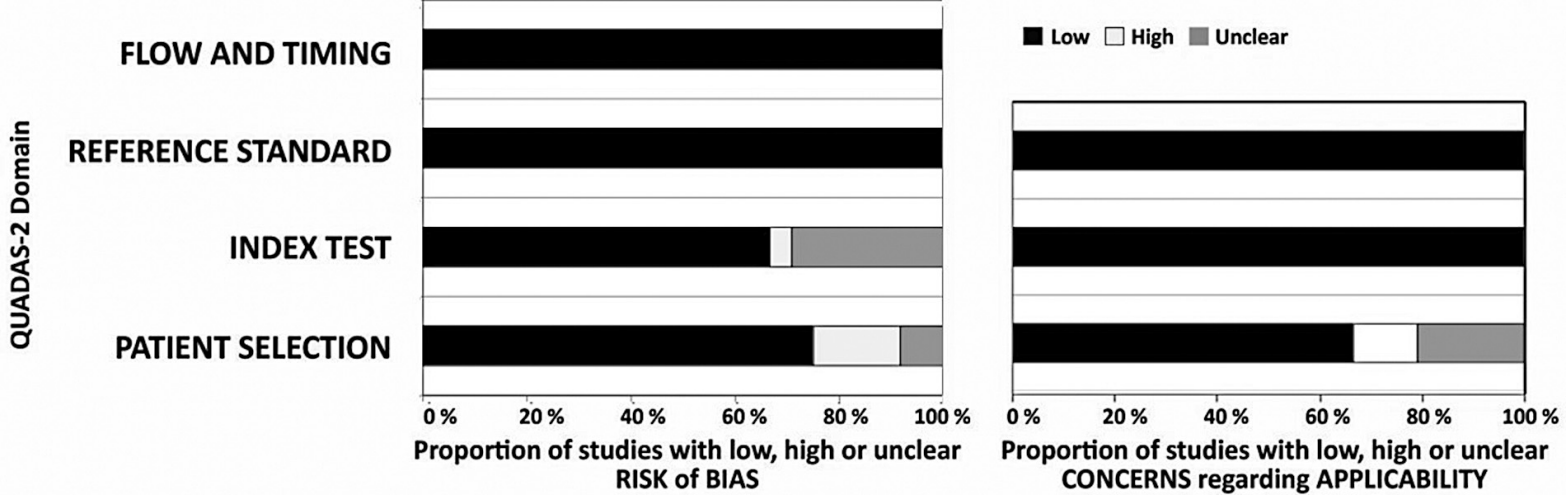
Summary results of QUADAS-2 analysis of all studies meeting inclusion criteria.

The QUADAS-2 analysis ( Fig. 2 ) showed the quality assessment results for the studies included in this systematic review. In the domain of patient selection, we identified unclear risk of bias in six studies that focused solely on metastatic lymph nodes (comprising 27% of the sample) or included primary tumors with metastatic lymph nodes 23 25 31 34 38 40 ; additionally, three studies were judged to have an unclear risk of bias because they did not specify whether patients were enrolled consecutively. 28 36 37 Furthermore, three studies were assessed as having a high risk of bias owing to a small sample size. 10 25 35 High applicability concerns were associated with studies that included both primary and some recurrently treated HNSCC cases. 22 39 41

In the domain of index test, unclear risk of bias emerged for seven studies not clearly describing if cystic or necrotic area were excluded during lesion segmentation 10 25 26 29 35 36 39 ; since 18F-FDG has a half-life of 109.7 minutes, high risk was attributed when PET/CT imaging acquisition occurred more than 300 minutes after radiotracer injection ( Table 2 ), potentially leading to SUV underestimation. 26

**Table 2:** Hardware and best functional parameters used to perform correlation between apparent diffusion coefficient and standardized uptake value in HNSSC

Year	Study	PET/CT		MRI			Interval between PET and MRI	Segm. technique		Best Correlation
		FDG (MBq)	Acq (min)	Tbp (min)	Field (T)	b-value (s/mm ^2^ )	Mean time (day)	PET (M/S)	MRI (M/S)	Functional parameters
2011	Fruehwald-Pallamar 8	300	50	3	3	0,800	14	M	S	NA
2011	Choi 36	5.2/kg	60	2	1.5	0,1000*	14	M	M	SUVmean vs ADCratio
2012	Nakajo 37	3.7/kg	60	NA	1.5	0,800	13	M	M	SUVmax vs ADCmean
2012	Nakamatsu 38	166–320	60	2	1.5	0,1000	20	M	M	SUVmax vs ADCmin
2013	Varoquaux 39	370	60	3	1.5; 3	0,1000	3.5	M	M	NA
2014	Ng 40	370	50-70	3	3	0,800	NA	M	S	NA
2015	Covello 41	406 ± 40	81 ± 15	3	3	0,500, 800	1, same time	M	M	SUV vs ADCmean
2015	Han 42	5/kg	60	2.5	1.5	0,1000	3.2	M	S	SUV vs ADCmean
2015	Gawlitza 22	5/kg	60–120	NA	3	0,800	1, same time	M	S	SUVmax vs ADCmin
2015	Martins 23	5.6/kg	60–120	NA	1.5	0,1000	1, same time	NA	NA	NA
2016	Surov 10	5/kg	60–121	NA	3	0,800	1, same time	M	S	NA
2017	Núñez 25	370 ± 37	60	NA	1.5	0, 600, 1000	NA	M	M	SUVmean vs ADCmean
2017	Leifels 26	4/kg	60-300	NA	3	0, 800	1, same time	M	M	SUVmax vs ADCmin
2017	Rasmussen 9	4/kg	100–120	1 bed, 20	3	0, 500, 1000	3	M	M	SUV vs ADC
2018	Dang 27	3.7–7.4 /kg	119-151	1 bed, 25	3	0, 800	1, same time	M	M	NA
2020	Cheng 28	2.9–3.7 /kg	50-70	2	3	0,50,200, 500,800, 1000*	NA	M	M	NA
2020	Çolak 11	3.7/kg	60	1.8	3	0,800	15	M	M	NA
2020	Zhang 29	3.7/kg	60	1, 10	3	0,800	14	M	S	MTV vs ADCmean
2021	Garau 30	3.7/kg	60	3	1.5	0,1000	13	M	S	SUVpeak vs ADCsd
2021	Paudyal 31	300–450	70–80	5	3	0,20,50, 80,200, 300,500, 800*	NA	M	S	SULmean vs ADC
2021	Bülbül 32	4/kg	60	1.5	1.5	0,800	10	M	M	SUVmax vs ADCmean
2022	Gupta 33	NA	NA	NA	NA	NA	NA	NA	NA	MTV vs ADCmean
2022	Freihat 12	NA	NA	1 bed	3	NA	1, same time	M	M	NA
2022	de Koekkoek-Doll 34	190-240	50-70	3	3	0,100, 200,300, 500,800, 1000	NA	M	M	SUVmax vs ADCmin
2023	Wongsa 35	2.5/kg	60	NA	3	0,800	1, same time	M	S	SUVmax vs ADCmean

Acq, Time elapsed from injection of radioactive drug to PET/CT imaging acquisition; ADC, apparent diffusion coefficient; ADCmean, mean apparent diffusion coefficient, expressed as × 10 − 3 mm ^2^ /s; ADCmin, minimum apparent diffusion coefficient, expressed as × 10 − 3 mm ^2^ /s; ADCratio, ADC values of tumor to normal tissue.; ADCsd, ADC standard deviation, expressed as × 10 − 3 mm ^2^ /s; b-value, degree of diffusion weighting applied; FDG, 18F-fluorodeoxyglucose; HNSCC, Head and neck squamous cell carcinoma; M, manual; MBq, Megabecquerels; MTV, metabolic tumor volume; S, semiautomatic; Segm. technique, segmentation technique; SULmean, SUVmean normalized to lean body mass; SUV, standardized uptake value, measuring tracer uptake in a lesion normalized to a distribution volume; SUVmean, average SUV; SUVmin, the lowest SUV in a given region of interest; SUVpeak, maximum average SUV within a 1 cm3 spherical volume; T, tesla; Tbp, time per bed position which increase the total scanning time.

* The authors reported also acquisitions using b-values greater than 1000 s/mm ^2^ (e.g., 1500, 2000 or 3000 s/mm ^2^ ); NA, not applicable

### Meta-Analysis: The Correlation Between ADC and SUV

**Fig. 3:**
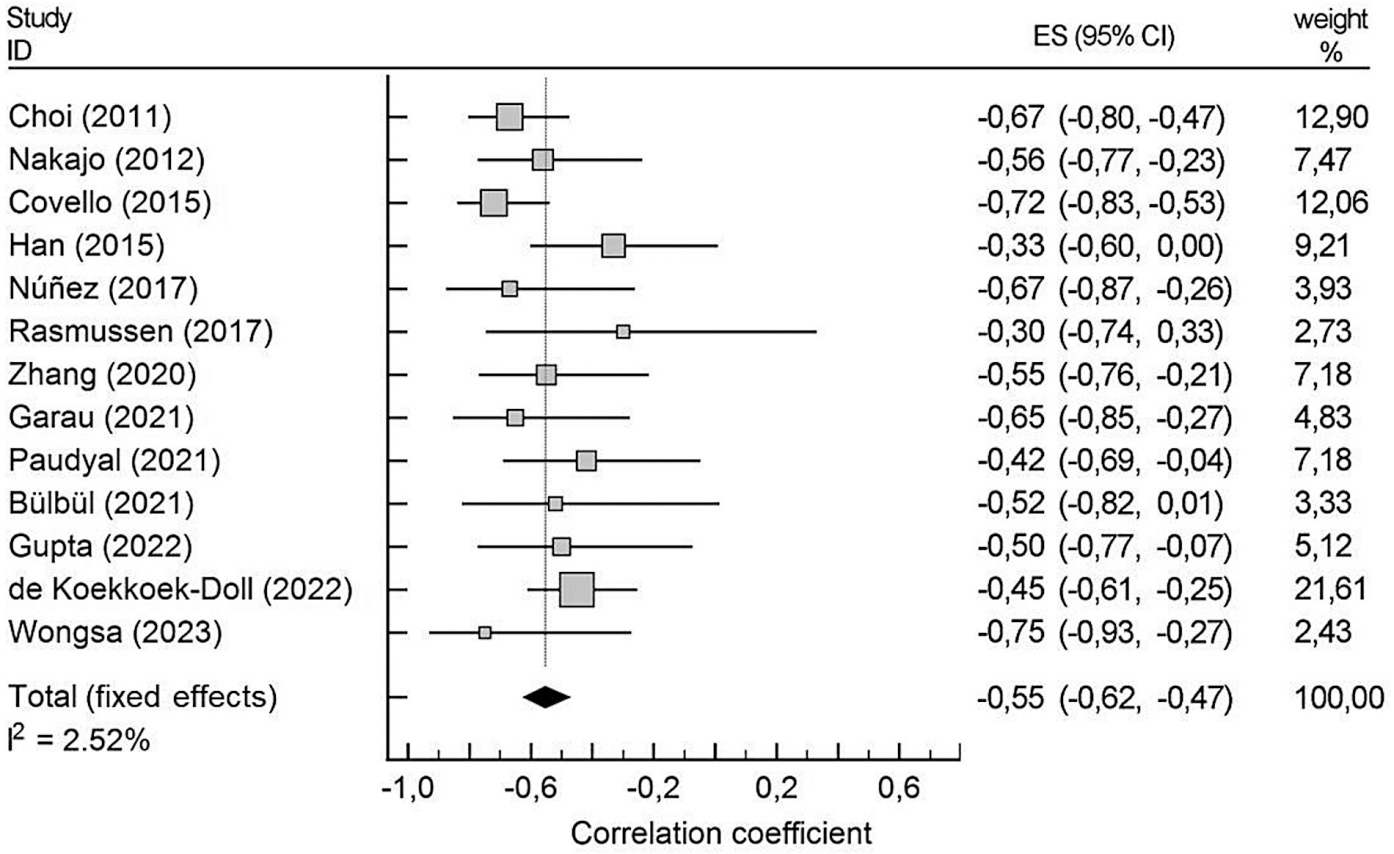
Plot of pooled values derived from correlations between apparent diffusion coefficient and standardized uptake value.

**Fig. 4:**
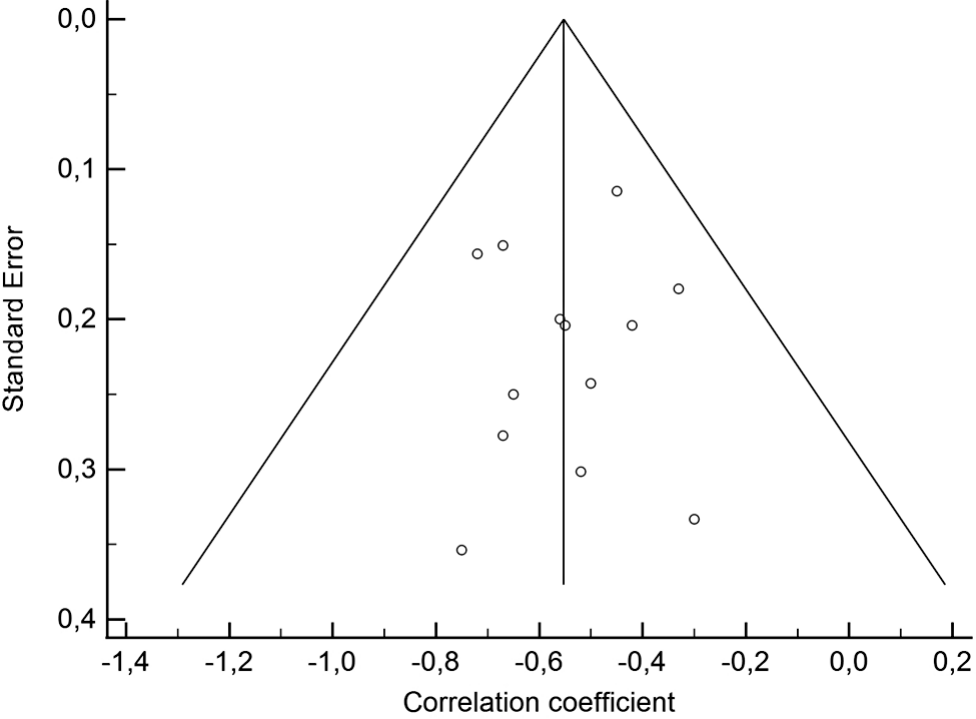
Funnel plots of the 13 studies included in this meta-analysis. For each effect size, data are plotted against their standard error. Vertical solid line inside the triangle indicated the summarized estimate of the effect size; oblique lines showed the 95% confidence limits around the summarized effect size.

Thirteen studies, including 367 (out of 790) patients, reported a statistically significantly correlation between ADCs and SUVs, with a pooled estimate of correlation coefficient of ρ = – 0.55 (95% confidence interval [CI], – 0.624 to – 0.473). Including additional data from two studies that observed a trend towards correlation between functional parameters, 22 26 the sample size increased to 418 patients ( Table 3 ). The I ^2^ index described and quantified the magnitude of heterogeneity, representing the part of total variation attributable to between-studies variance. 43 Notably, a low I ^2^ index of 2.5% emerged ( Fig. 3 ). The summarized ρ value and I ^2^ index did not change substantially even with the inclusion of two studies 22 26 reporting a trend toward correlation (P = 0.008, for a total of 51 patients). Due to the limited sample size and low heterogeneity, subgroup analyses were not conducted. Finally, the corresponding funnel plots indicated that most research results were close to axis, demonstrating the absence of publication bias in these studies ( Fig. 4 ).

**Table 3:** Distribution of correlation between ADC and SUV based on clinical characteristics

Clinical characteristics	Patients without correlation between ADC and SUV % (No.)	Patients with correlation between ADC and SUV % (No.)
**Gender**		
Men	51 (257)	49 (245)
Woman	58 (75)	42 (54)
**Site**		
Oral cavity	28 (63)	72 (99)
Oropharynx	36 (74)	64 (138)
Hypopharynx	35 (85)	65 (48)
Rhinopharynx	27 (58)	43 (44)
Sinus	21 (4)	79 (15)
Larynx	25 (33)	75 (55)
Other	15 (4)	85 (18)
**Staging**		
T1	41 (23)	59 (33)
T2	61 (59)	39 (39)
T3	71 (87)	29 (36)
T4	67 (94)	33 (45)
**Grading**		
G1 + G2	33 (47)	67 (77)
G3	42 (79)	58 (111)
**Overall** **ADC-SUV correlation**	48 (372) *	52 (418) *

G1 + G2, well to moderately well differentiated HNSSC; G3, HNSSC with low grade of differentiation; HNSCC, Head and neck squamous cell carcinoma; No., number of patients.

* Including Leifels and Gawlitza studies 22 26 showing a trend toward correlation (P = 0.08).

## Discussion

Fifty-two percent of the sample showed an inverse significant correlation between water diffusion restriction and glucose uptake in HNSCC. Given the degree of correlation, which reached a moderate intensity of ρ = – 0.55 (95% CI, – 0.624 to – 0.473; P < 0.05), these functional parameters appear to exhibit a certain degree of connection. This finding may be explained as follows: the viable tumoral area with increased cellular glucose metabolism also demonstrates reduced extracellular Brownian motion of water molecules because of high tumoral cellularity.

Previously, meta-analyses by Deng and Shen identified weak negative correlations between ADC and SUV values in HNSCC, with correlation coefficients of ρ = −0.31 (95% CI: – 0.44 to – 0.19) and ρ = – 0.27 (95% CI: −0.39 to −0.15) 15 44 ; however, both analyses included only six studies, whereas this meta-analysis summarized a larger sample size to provide more robust and reliable results. Consistently, it has been shown that ADC correlated well with cellular density and nuclear cytoplasmic ratio in different epithelial malignancies, such as prostatic cancer, renal cell carcinoma, laryngeal and hypopharyngeal carcinomas. 24 45 Similarly, several studies reported a correlation between tumor fluorodeoxyglucose uptake and markers of cell proliferation, such as DNA replication in the S phase or Ki-67 expression. 46 47 Furthermore, an inverse relationship between SUV and standard deviation of ADC (ADCsd) is noteworthy. 30 The ADCsd reflect intra-tumor variability in cell proliferation, offering insights into regions where water diffusion is restricted or facilitated based on tissue characteristics. This finding suggested that uniform HNSCC tissue may be associated with higher glycolytic activity and a more aggressive phenotype, while post-treatment changes, such as inflammation, fibrosis, and proteinaceous fluid, could lead to heterogeneous tissue with broader diffusion restriction values and reduced glucose uptake, potentially influenced by macrophage activity. 5 48 49

On the other hand, not all studies in this systematic review confirmed these findings. It is plausible that various factors, ranging from molecular characteristics to clinical settings, influenced the relationship between water diffusion restriction and glucose uptake. Such factors could include sample characteristics, operator variability, acquisition protocols, and hardware configurations.

Regarding sample characteristics, a high percentage of moderately or very advanced (T4) nasopharyngeal tumors exhibited no correlation between ADC and SUV. These cases were more likely to present necrosis due to the aggressive nature of the tumors, compared to earlier-stage HNSCC. Necrotic, non-metabolic tissue may restrict water diffusion, yielding lower ADC values, while exhibiting mild surrounding metabolic activity due to inflammatory processes. Conversely, an inverse correlation between ADC and SUV was observed in a significant proportion of early-stage oro-oropharyngeal cancers, likely due to the better preservation of functional characteristics of the tumor tissue. 34 36

Unfortunately, this meta-analysis evaluated the correlation between ADC and SUV without accounting for tumor differentiation grade due to insufficient data. However, the possibility that glucose uptake and hypercellularity are closely linked in more undifferentiated lesions cannot be ruled out, as widely reported in several tumors. 50 51 52 53 54

Importantly, operator variability in manual placement of regions-of-interest (ROIs) and lesion segmentation techniques could have influenced the correlation between ADC and SUV. 55 In particular, the inclusion of hypo-metabolic or non-metabolic areas, both hypo-restricted (cystic) and/or hyper-restricted (necrotic), alongside viable tumor tissue during segmentation, may have affected ADC values. 10 25 35 36 For instance, ROIs drawn along tumor borders may encompass segmented tissue with high or low ADC value associated with a low or absent metabolic activity. In contrast, the calculation of metabolic activity by semiautomatic thresholding could be less dependent on operator variability and tumor composition compared to manual method segmentation. 10 23 29 30 31 35 37 42

Differences in imaging protocols and instrumentation likely also influenced the findings. PET/MRI systems enable the acquisition of metabolic activity and diffusion-weighted data in the same session, reducing spatial and temporal discrepancies between the two imaging modalities and ensuring a more reliable comparison between SUV and ADC. 35 By contrast, studies where PET and MRI were acquired separately often involved a time delay of up to twenty days (as observed in this meta-analysis) between scans. 38 During this period, necrosis with potentially associated colliquative phenomena and variation in metabolic activity may occur, confounding the correlation between SUV and ADC.

The understanding of the interplay between ADC and SUV may provide valuable insights for tumor management. During early detection, the SUV-to-ADC ratio could potentially indicate lesion aggressiveness, allowing for more targeted interventions. 10 Biopsy guidance might also be improved by prioritizing areas with restricted diffusivity and higher radiopharmaceutical uptake, which could enhance diagnostic accuracy. Moreover, the integration of ADC and SUV as imaging biomarkers may aid in stratifying patients based on tumor aggressiveness, potentially guiding tailored treatment strategies. 54 Additionally, incorporating diffusion- and metabolism-based imaging in radiotherapy planning might refine tumor contouring and spare healthy tissue, reducing side effects. 31 Changes in the relationship between ADC and SUV during therapy could finally serve as indicators of tumor response; monitoring a potentially increasing ADC (reflecting tumor structural changes with reduced cellular density) alongside a decrease in metabolic activity may also facilitate tailoring interventions to individual response profiles.

Our study has several limitations. First, the exclusion of papers reporting non-significant correlations between ADC and SUV, necessitated by the statistical method employed, may have limited the pooled analysis. 18 19 However, it provided a quantitative benchmark that future studies on the same topic can test in biologically homogeneous sub-groups; moreover, we thoroughly analyzed all studies identifying factors that might explain the lack of correlation (e.g., advanced-stage tumors with distinct environmental characteristics) to provide a balanced interpretation. Secondly, the variability in tumor populations across studies may have introduced bias; for instance, HNSSC can exhibit distinct genomic features based on their etiology, such as HPV-positive tumors being associated with TRAF3 alterations, while HPV-negative and smoking-related tumors are characterized by TP53 mutations and CDKN2A inactivation; such genetic variability may affect tumor behavior and imaging biomarkers, potentially influencing the observed correlations in this meta-analysis. 56 Furthermore, other sources of error may be present, such as the partial volume effect, which may have altered functional values, especially for small lesions, weakening the correlation between SUV and ADC. The variability in imaging instrumentation, acquisition protocols, segmentation method and MRI to PET/CT interval likely contributed to inconsistencies in the observed functional values or correlation, and accounted for part of the between-study variability; for instance, the wide range of b-values across studies (with value exceeding 1000 s/mm ^2^ in three studies), as higher b-values emphasize restricted water diffusion and yield lower ADC values. 28 31 36 However, because all ADC values within a given study were proportionally affected, their relative distribution remained nearly unchanged, with correlation between ADC and SUV expected to remain stable. Finally, the relatively small sample in some studies limited the reliability of their findings.

## Conclusion

This systematic review examined the relationship between functional parameters obtained from DW MRI and 18F-FDG PET/CT in patients with HNSCC. Although a significant inverse correlation between cellular proliferation and glycolytic activity was observed in many patients, this relationship was not consistently reported across all papers. This inconsistency highlights the need for caution in interpreting these findings. Future studies should focus on more homogeneous samples with consistent morphological and molecular characteristics to ensure greater comparability and robustness of results. Moreover, we encourage future investigations to adopt harmonized DWI/PET acquisition parameters and protocols, so that true modifiers can be isolated without confounding technical variability. Nevertheless, the statistical correlations observed suggest that leveraging the relationship between ADC and SUV, including their reciprocal variations, could provide additional context to better understand tumor biology. Exploring these dynamic interactions in clinical practice may offer preliminary insights into diagnostic and prognostic evaluations, potentially contributing to the identification of patients who might benefit from tailored therapeutic strategies.
